# Detection of airborne wild waterbird-derived DNA demonstrates potential for transmission of avian influenza virus via air inlets into poultry houses, the Netherlands, 2021 to 2022

**DOI:** 10.2807/1560-7917.ES.2024.29.40.2400350

**Published:** 2024-10-03

**Authors:** Alex Bossers, Myrna MT de Rooij, Isabella van Schothorst, Francisca C Velkers, Lidwien AM Smit

**Affiliations:** 1 Population Health Sciences - Institute for Risk Assessment Sciences, Utrecht University, Utrecht, The Netherlands; 2 Wageningen Bioveterinary Research, Wageningen University and Research, Lelystad, The Netherlands; 3 Population Health Sciences - Farm Animal Health, Utrecht University, Utrecht, The Netherlands

**Keywords:** avian influenza, wild birds, metabarcoding, environmental DNA, eDNA, air, particulate matter

## Abstract

**Background:**

Outbreaks of highly pathogenic avian influenza (HPAI) on poultry farms and in wild birds worldwide persists despite intensified control measures. It causes unprecedented mortality in bird populations and is increasingly affecting mammalian species. Better understanding of HPAI introduction pathways into farms are needed for targeted disease prevention and control. The relevance of airborne transmission has been suggested but research involving air sampling is limited and unequivocal evidence on transmission routes is lacking.

**Aim:**

We aimed to investigate whether HPAI virus from wild birds can enter poultry houses through air inlets by characterising host materials through eukaryote DNA sequencing.

**Methods:**

We collected particulate matter samples in and around three HPAI-affected poultry farms which were cleared and decontaminated before sampling. Indoor measurements (n = 61) were taken directly in the airflow entering through air inlets, while outdoor air samples (n = 60) were collected around the poultry house. Positive controls were obtained from a bird rehabilitation shelter. We performed metabarcoding on environmental DNA by deep sequencing 18S rRNA gene amplicons.

**Results:**

We detected waterbird DNA in air inside all three, and outside of two, poultry farms. Sequences annotated at species level included swans and tufted ducks. Waterbird DNA was present in all indoor and outdoor air samples from the bird shelter.

**Conclusion:**

Airborne matter derived from contaminated wild birds can potentially introduce HPAI virus to poultry houses through air inlets. The eDNA metabarcoding could assess breaches in biosecurity for HPAI virus and other pathogens potentially transmitted through air via detection of their hosts.

**Key public health message**

**What did you want to address in this study?**

Highly pathogenic avian influenza (HPAI) virus (bird flu) affects mainly poultry and wild birds, but bird-to-human transmission can also occur. Poultry farms in the vicinity of infected waterbirds are at increased risk, possibly by the airborne introduction of HPAI. The virus itself is difficult to measure in the air. Alternatively, we tried to detect genetic material (DNA) from waterbirds (which may carry the virus) in the air that flows into poultry farms.

**What have we learnt from this study?**

This study indicates potential airborne introduction of HPAI virus into poultry farms. We found particles containing waterbird DNA in the airflow entering farms. It is therefore likely that also HPAI virus could be introduced via the same route together with materials from infected waterbirds. Our approach can be extended to other pathogens and/or animals to elucidate routes of transmission.

**What are the implications of your findings for public health?**

Using the example of HPAI virus, this study highlights potential airborne entry of important pathogens via air inlets into farms. With better understanding of transmission, biosecurity measures can be implemented to strengthen disease prevention at farms. Given the interconnectedness of humans, animals and the environment, enhanced infectious disease control in livestock will decrease the livestock-associated disease burden in humans.

## Introduction

The introduction of pathogens from wild animals into domesticated or farmed animal populations is an important global issue. From 2016 onwards, outbreaks of highly pathogenic avian influenza (HPAI) in poultry farms in Europe have been recurring [1-3]. Infections with HPAI viruses have also caused unprecedented mortality in wild bird populations and increasingly affects mammalian species [4]. The main current prevention and control measures for HPAI in poultry in Europe consist of strict biosecurity measures and, in farms affected by an outbreak, culling of all birds. Presence of wild birds near poultry farms, in particular *Anseriformes* (ducks, geese and swans), is associated with increased risk of HPAI introduction in poultry [5,6]. Compliance with biosecurity measures and avoiding direct contact between poultry and wild birds by obligatory indoor housing reduces the risk of HPAI introduction into the flock. However, many indoor-housed poultry on farms, also on farms with apparently high biosecurity standards, have become infected, indicating that transmission routes of HPAI from wild birds to indoor-housed poultry are still poorly understood [7,8]. Entry via air inlets of airborne HPAI-contaminated particles derived from nearby infected wild birds could be a relevant route.

Avian influenza is able to survive in outdoor environments for periods of a few months at ideal conditions but for shorter periods (up to 7 days) around room temperature [9]. Feathers can be infectious or easily become contaminated with virus from faecal particles and can function as fomites. Airborne HPAI dispersal may thus be important and potentially play a role in introducing the virus into a flock. This has been indicated by several studies on transmission between farms at close distances but might also be relevant for initial HPAI virus introduction into the farm from infected waterbirds nearby [10,11]. Investigating this potential airborne route of introduction by targeting for the HPAI virus in air entering the houses is highly challenging. This requires air sampling during the presence of wild birds shedding avian influenza virus in the proximity of the farm [7], resulting in a narrow time window with low probability of virus detection considering also relatively low viral loads. Alternatively, capturing host-derived biological materials such as small feathers and faeces particles in air flows entering farms can be used to highlight potential entry pathways of host-associated pathogens. Initial approaches thus far, employing nets, showed that relatively larger materials (several mm) can enter through air inlets. Mainly insect and plant materials were observed visually, but wild waterbird materials such as feathers were not seen [12]. To gain better insight, more advanced sampling and analysis approaches are needed to characterise airborne particle transmission.

**Figure 1:**
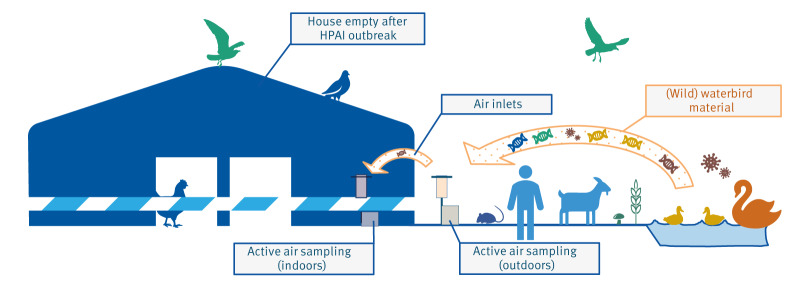
Detection of wild bird eDNA in air inside and outside of a poultry farm

In this study, we applied environmental DNA (eDNA) metabarcoding (deep sequencing) to the context of infectious disease epidemiology. Metabarcoding has been applied before in biodiversity research; the few studies performed showed that a large diversity of eukaryotic species could be detected simultaneously and over larger distances in airborne particles [13,14]. Here we aimed to determine whether wild waterbird DNA can be detected in the airflow entering poultry houses (Figure 1). Since the probability of detecting HPAI virus itself in air samples is low, we targeted its wild host species as an indicator for potential HPAI transmission through air.

eDNA: environmental DNA; HPAI: highly pathogenic avian influenza.

The poultry house was cleared and disinfected after an HPAI outbreak occurred before the time of sampling. Active air sampling was performed in and around the poultry farms. Potential sources of environmental DNA in addition to *Gallus gallus* (chicken) and *Anseriformes* (waterbirds) are indicated.

## Methods

### Locations

We performed outdoor and indoor air sampling at three poultry farms in the Netherlands, two broiler farms (B1 and B2) and one layer (L) farm. We selected these farms based on farmers’ willingness to participate and a recently experienced HPAI outbreak. These farms tested positive for HPAI virus subtype A(H5N1) at the end of 2021 or early 2022. At that time, all poultry was housed inside, and expert evaluation of these farms indicated a high biosecurity standard [15]. According to the regulations of the national competent veterinary authority (Netherlands Food and Consumer Product Authority (NVWA)), poultry on affected farms need to be culled, and thereafter follows a long and intense procedure with thorough cleaning and repeated disinfecting. This provided us with a unique opportunity to investigate air in and around farms located in areas with high HPAI risk at a time when no poultry flocks were housed, thus avoiding interference of our sampling with normal farming practices. At the time of the measurements, the broiler houses were completely empty. At the layer farm, the house contained 25 sentinel chickens in Compartment 1 during the first cycle of measurements performed in Compartments 2 and 3. All three poultry farms were located in waterfowl-rich regions, as described previously [12]. We also collected air samples as positive controls at a bird rehabilitation shelter (S), housing a variety of waterfowl and other birds both in indoor (first phase of care) and outdoor aviaries (later phase of recovery).

### Air sampling

At each location, we repeatedly collected indoor and outdoor air samples. Air sampling equipment was used in analogy with earlier studies in and around livestock farms [16-18]. Teflon filters were used to collect air samples (total suspended particles (TSP)) over 4–5 consecutive days (one measurement cycle) by Harvard impactors operating at a flow of 10 L/min (ca 65 m^3^ of air sampled per filter). After each measurement cycle, we immediately collected the filters and stored these on the same day at −80^o^C. Per measurement cycle, we also collected one indoor field blank and one outdoor field blank to assess potential (cross-) contamination issues. Field blanks are unexposed filters (not connected to the sampling pump) that underwent a similar handling as the exposed filters.

At the three poultry farms, we performed outside air sampling around the poultry house in each of the four wind directions (north, east, south, west) at close distances to the farm (between 12 and 25 m depending on local situation and practicalities e.g. avoiding pathways and ditches). Inside the poultry houses, we positioned the air sampling installations to sample directly the air flowing in through air inlets; an impression of the sampling positions outside the poultry houses as well as inside the poultry house in the direct air flow from the air inlet, is appended in Supplementary Figure S1. During the sampling period, the mechanical ventilation system (regulated by a computer) was programmed such that the air flow through air inlets was stabilised to represent normal operational conditions with a flock housed in the farm.

### Nucleic acids isolation

We extracted DNA and RNA from the filters following a low biomass protocol [19]. Empirically (data not shown), we demonstrated using DNA and RNA virus spike-in experiments that RNA from viruses could be isolated with great efficiency as well, making the RNA extracts suitable for measurements by qPCR (HPAI diagnostics). We included extraction blanks and field blanks for each sampling round and each batch of DNA and RNA extractions. Extractions of positive controls were handled in the last separate batch to avoid cross-contamination.

### Metabarcoding and deep sequencing

We selected PCR primers 18Sa_F 5’-ATAACAGGTCTGTGATGCCCT-3’ and 18Sa_R 5’-CCTTCYGCAGGTTCACCTAC-‘3 to target the hypervariable regions V8–V9 from the 18S rRNA gene [20]. Amplification for 25 cycles, indexing for six cycles and sequencing on an Illumina MiSeq sequencer was performed as described for 16S [21], targeting an 18S amplicon sequencing depth > 100,000 paired-end 300 bp sequence clusters per sample.

### Data analysis

Raw sequencing data were primer-clipped, deblurred, error-corrected and annotated using version 1.26.0 of the dada2 R-package [22] at default settings except for *truncLen*=(190,180), *minOverlap*=10, *maxN*=2, *maxEE*=2, *minFoldParentOverAbundance*=2, chimeraMethod consensus, and the dada2 pseudo pooling strategy. We subsequently annotated amplicon sequence variants (ASVs) with the dada2 naïve Bayesian classifier and a custom-built 18S sequence database containing all 1,252 sequences in National Center for Biotechnology Information (NCBI) non-redundant (NR)/nucleotide database (accessed: 27 Oct 2022) from the taxonomic classes *Aves* (n = 265) and *Mammalia* (n = 987). We replaced non-available taxonomic rank values with the first known higher taxonomic rank value using a prefix for the taxonomic rank it originated from. We created a phylogenetic maximum likelihood neighbour-joining tree from the detected ASVs using mega-x version 11 [23] which we further curated manually. The evolutionary history of ASVs was inferred by using the maximum likelihood method and Tamura–Nei model.

### Highly pathogenic avian influenza virus RNA qPCR

To enable comparisons between our novel eukaryote DNA sequencing approach and traditional direct pathogen detection, we additionally performed qPCR analyses to detect avian influenza virus RNA in available duplicate air samples (n = 15). An accredited diagnostic qPCR targeting the M segment of the avian influenza genome (Wageningen Bioveterinary Research) was used with a detection limit at 10–100 virus particles as estimated from spike-in benchmark values under the used practical total nucleic acid isolation conditions [1].

## Results

### Genetic material in air samples

At the three poultry farms, we detected DNA of waterbirds in four of the 47 indoor air samples collected on the three farms (B1, B2, L) and in three of the 52 samples collected outside at two farms (B2, L). These waterbird DNA-positive air samples (n = 7) were taken at various time points and locations in and around the farm (Table); in the Supplementary Table, we provide more details on timing and location of these positive samples. Waterbird DNA was present in all indoor and outdoor air samples collected at the bird shelter. 

**Table:** Sampling programme, collected air samples and samples positive for wild waterbird DNA, by time period and location type, the Netherlands, December 2021–April 2022 (n = 119)

Location		B1	B2	L	S
Type		Broiler farm	Broiler farm	Layer farm	Bird shelter
Sampling	From	10 Dec 2021	15 Jan 2022	10 Feb 2022	23 Mar 2022
	Until	24 Dec 2021	7 Feb 2022	14 Mar 2022	19 Apr 2022
Indoor	Cycles (every 4–5 days)	1	5	5	6
	Positive/negative samples	1/5	1/21	2/21	12/12
Outdoor	Cycles (every 4–5 days)	3	5	5	4
	Positive/negative samples	0/10	1/21	2/21	8/8

Sequencing the 18S-derived amplicons from all 119 air samples resulted in a median of 126,617 (range: 11,538–294,079) paired-end sequencing reads per sample. After dada2 processing and chimera filtering, this revealed a median of 37,314 (range: 17,200–150,990) annotated ASVs per air sample with the majority (87.1%) between 330 and 335 bp long. In total, we detected 54,436 different ASVs; they were a good representation of the sampled community (probability of completeness by Good’s coverage estimator on singletons of 0.99689). All field blanks were negative for ASVs belonging to the orders *Anseriformes, Passeriformes, Rodentia,* or other closely related species, indicating absence of cross-contamination with these species from any of the control samples in the procedures. 

In the additional qPCR analyses performed, all tested samples were negative for avian influenza virus RNA, indicating that the virus was either absent or present in quantities below the limit of detection.

### Overview of *Aves* amplicon sequence variants detected in air samples

**Figure 2:**
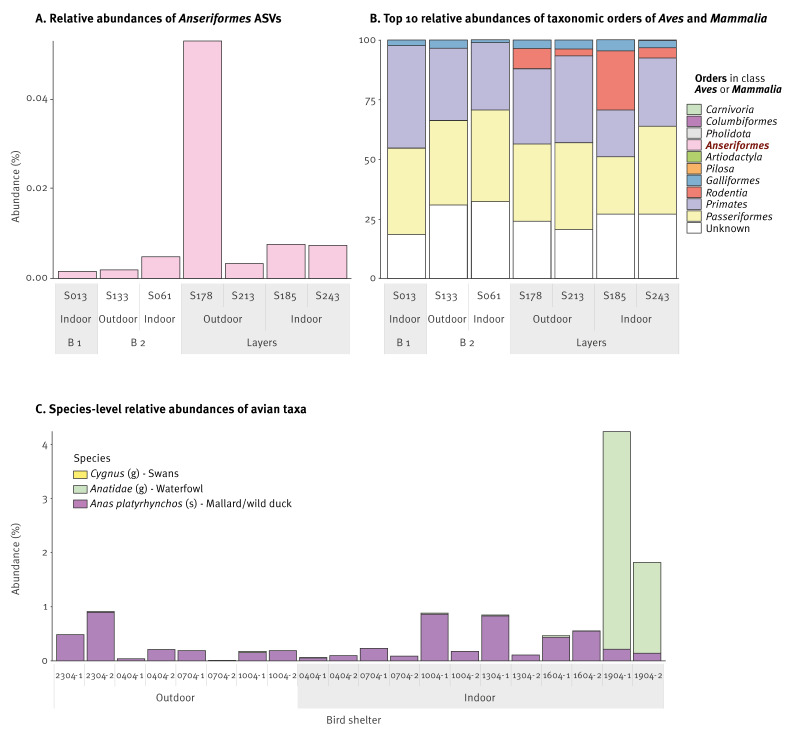
Relative abundances of *Anseriformes*-positive air samples from poultry houses and the bird shelter at taxonomic order level, the Netherlands, December 2021–April 2022 (n = 7 and 20 respectively)

Figure 2 shows the relative abundance in our samples of *Anseriformes* ASVs within all annotated *Aves* and in addition to mammalian taxa. There was clear variation in the relative abundances of waterbird DNA in the seven positive air samples collected at the three poultry farms (Figure 2A). An overall impression of the classes *Aves* and *Mammalia* for the same samples is depicted in Figure 2B. This shows that we also captured DNA of other animal species, including other wild birds (i.e. *Passeriformes* and *Columbiformes*), chickens and rodents, warranting follow-up in future research. Figure 2C shows that all positive control samples collected at the bird shelter contained *Anseriformes* DNA, with varying relative abundances including expected visually observed species (data not shown).

Relative abundances in panel B are shown as stacked bar graph sorted on highest relative abundance (very low abundant taxa are barely visible).

### Phylogeny of a representative selection of detected amplicon sequence variants

**Figure 3:**
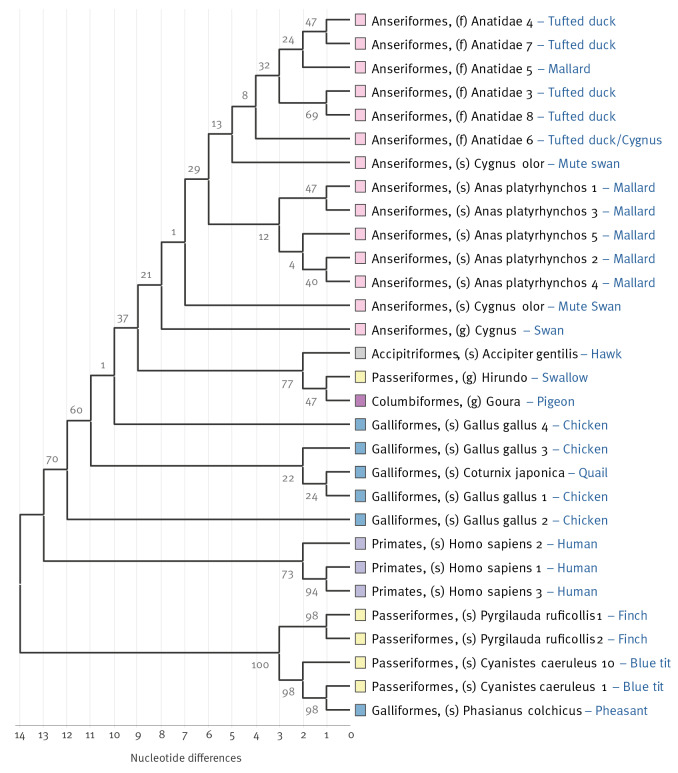
Maximum likelihood phylogenetic tree of detected amplicon sequence variants^a^ from the order *Anseriformes* and selected contrasting/phylogenetically close species sampled at poultry houses, the Netherlands, December 2021–April 2022

^a^ Hypervariable regions V8−V9 of the 18S rRNA gene.

Figure 3 shows the phylogenetic tree constructed from all detected ASV sequences belonging to the waterbirds (order *Anseriformes*), supplemented with detected species that are phylogenetically close (orders *Passeriformes, Accipitriformes*, *Galliformes* and *Columbiformes*) as well as several detected human ASVs. The tree with the highest log likelihood (−1,683), is shown. This analysis involved 30 nucleotide sequences. There were a total of 340 aligned positions in the final dataset. The inferred tree shows that detected waterbird ASVs were at least 18 nt (from the median 333 bp ASV length) different from the other closest orders (Figure 3). *Anseriformes* sequences clustered mostly at the species level, while ASVs from other orders clustered more scattered throughout the tree at higher taxonomic levels.

Taxonomic classification at order level where coloured squares correspond to the various classifications. Order classification is followed by the lowest assigned taxonomic level family (f), genus (g) or species (s). Numbers in the species name indicates the nth sequence from the taxonomy table of detected amplicon sequence variants. Species common names are indicated in blue at the end. Number of nucleotide differences between ASVs are indicated as well as the bootstrap values (%) at each branching point.

## Discussion

This study showed, through innovative application of eukaryote eDNA metabarcoding, that wild waterbird materials were present in the airflow entering poultry farms and also around poultry farms. Detecting these biological materials from potential HPAI hosts in the air flowing via air inlets into the poultry farms indicates that HPAI could be introduced in the flock through this airborne route and subsequently lead to an outbreak. This potential route of entry could be an explanatory factor in the surge of outbreaks in poultry farms caused by HPAI virus introduction after an increase in the presence of infected wild waterbirds. The DNA barcoding method would allow assessment of compromised biosecurity and effectiveness of potential intervention strategies without the need to capture HPAI at the time of sampling.

Research on the epidemiology of HPAI has intensified over the years, especially as conventional control measures appear insufficient to limit the number of affected farms [3,8]. Outbreaks in flocks still occur frequently, despite extended periods of mandatory indoor housing and enhanced biosecurity. Measures are aimed at limiting direct and indirect contact of domestic poultry with wildlife. Wild waterbird populations are known reservoirs of avian influenza viruses. They can distribute new strains through migration over long distances and can facilitate recombination of new strains in migratory and resident bird populations, being a source of outbreaks in domestic poultry worldwide. The actual host range for avian influenza is broad, but wild birds are regarded as the most important hosts introducing the virus into domestic flocks especially when water-rich environments are in the vicinity of farms [5,6]. 

Studies on HPAI characteristics indicate efficient spread of the virus through the environment on water, dust or larger particles [10,24,25]. Research on HPAI strain genome variability in the outbreak in the Netherlands in 2020 and 2021 indicated HPAI from wild birds ranging near poultry farms as the most obvious origin of the virus introduced in poultry houses but did not unequivocally indicate the actual transmission route [3]. Attempts to detect contact between wild birds and chickens using a microbial proxy or changes in the cloacal microbiota of chickens that have free access to an outdoor range largely failed [21]. Furthermore, initial attempts using nets over the air inlets at three poultry farms did not capture visible wild bird-derived materials such as feathers [12]. Another recent study was able to detect HPAI virus in air collected inside and outside at the time when clinically affected animals were present in the poultry houses [7]. Investigating wild bird transmission via detection of wild bird-derived DNA in a surveillance context has been suggested before but has as yet not been implemented [8]. Our study, which combined air sampling with eukaryote DNA metabarcoding, demonstrates that airborne wild bird DNA-containing materials can actually enter the poultry farms via the active airflow through the air inlets under normal commercial operating conditions.

Among bacteria and archaea, the ribosomal 16S rRNA gene is typically used as a universal barcoding gene to determine the quantity and taxonomic classification up to species level. The eukaryotic barcoding genes typically include the ribosomal 12S or 18S rRNA genes, the cytochrome oxidase I gene (COI/COX) or ribosomal inter-spacer regions ITS1 or ITS2 [26]. Similar to 16S rRNA gene amplicon sequencing, as used in microbiota research, eukaryotic markers are increasingly used to assess fungal composition mostly up to genus level and some to species level, using the well curated UNITE database [27,28]. Inter-spacer regions can also be used for other non-fungal species, however, the non-availability of large collections of well curated annotated ITS sequences including the taxonomic class *Aves* does not allow this. Despite the large barcoding-of-life initiatives targeting single species using the COI/COX gene, the sequence length and variability does not optimally support metabarcoding strategies by short-read deep sequencing strategies yet. Other methods using shotgun metagenomics sequencing or mitochondrial DNA reconstruction (Huanan market study trying to resolve the originating host-species of COVID-19 [29]), are also feasible but typically restricted to single-species classification strategies. Even though 12S rRNA barcoding has been applied for mammals and birds in airborne dust samples collected at a zoo [13], it tends to be fairly domain-specific depending on the PCR primer sets used. Since only taxonomically accurately annotated 12S rRNA sequences are limited, especially for the *Anseriformes* (waterbird) order, we selected sequencing the hypervariable regions V8–V9 from the 18S rRNA gene.

For 18S rRNA, several dedicated taxonomic databases exist (such as the highly curated SILVA database), but at the moment of writing, the coverage of the class *Aves* was relatively low, while the NCBI NR/nucleotide database had a better coverage of these species. We specifically selected primers spanning hypervariable regions V8–V9 [20] since these were most optimal in qPCR at our laboratories on a variety of samples from various species and environments, compared with primers targeting regions V4–V5 (data not shown). Furthermore, we refined the dada2 protocol for using 18S rRNA amplicons to generate longer ASVs. Consequently, we were able to assign taxonomy mostly at species level, stepping up to higher taxonomic levels when the particular ASV sequence could not be assigned at species level without conflict. Clustering sequences of 18S rRNA amplicons [30] was avoided to limit losing classification resolution. The phylogenetic tree demonstrated that keeping sequences at ASV level made the overall assessment for the order *Anseriformes* within the class *Aves* accurate enough for the current study. Prior ASV clustering below 97% identity would have lost this order-separating resolution. To substantiate the accuracy, we found that the diversity of visually observed bird species presence inside and outside the bird shelter reflected the detected eukaryote DNA diversity (data not shown).

We detected DNA of *Anseriformes* in all air samples collected at the bird rehabilitation centre and in at least one of the air samples collected at each farm. The findings at the poultry farms indicate variability of waterbird DNA in air over time (presence and/or load). Even though the Good’s coverage indicator indicates that we sequenced deeply enough to capture most of the present variability in species, rarefaction curves of the annotated sequences per sample suggest that for roughly half of the samples, deeper sequencing would have been beneficial; for additional data linking sequencing depth per sample to the detected diversity within these samples, we refer to the appended Supplementary Figure S2. Nevertheless, we did detect waterbird DNA, albeit at low amounts. Increasing the sequencing depth will increase the amounts of detected *Anseriformes* DNA increasing its overall sensitivity but will most probably not allow accurate quantification of the amounts of waterbird DNA due to large variation in gene copy numbers between species, cell types or even between single cells. Using a mitochondrial marker such as 12S or COI would most probably increase this problem because of the large variation in the number of mitochondria present per cell(type). For the current study, we are confident that we detected *Anseriformes* DNA, but we must be careful in interpreting our results beyond semi-quantitative categories (high, low and absent/not detected).

We purposefully selected three poultry farms considered at higher risk for virus introduction from wild bird populations for a proof-of-concept of our approach. These farms had recent outbreaks of HPAI, were located in water-rich areas and had substantial HPAI-confirmed wild bird mortalities in the vicinity; they were not selected to be representative of all poultry farms in the Netherlands. As a current limitation, we assume that airborne spread of avian influenza virus will typically be possible through virus-loaded biological materials from hosts, such as small particles of feathers and faeces. We expect the host DNA to be more stable than HPAI viral RNA in the environment, but this has not been investigated yet. As a follow-up study, linking the detection of eukaryotic DNA to the systematic visual observation of wild birds, mammals, rodents and vegetation around poultry houses is a logical next step to further substantiate our claims.

We carefully assessed whether derived sequencing reads could be the result of PCR or sequencing errors. However, the detected *Anseriformes* ASVs were at least 18 nt differences apart to the next closest species (20 nt differences for chicken), therefore we are confident that the detected *Anseriformes* DNA originated from waterbirds and were true positives. However, considering sensitivity, it could be that we have missed other potentially present eukaryotic species due to restrictions in sequence annotation limited by incomplete annotation databases.

We have demonstrated that the eukaryotic DNA metabarcoding approach can be used to detect host-derived materials in air in the context of HPAI and wild waterbirds. This approach can also be extended to other infectious agents and their corresponding hosts for investigating its transmission. This approach avoids the need to actually detect a certain pathogen travelling by air at the precise moment of introduction. It also provides direct semi-quantitative data for source attribution modelling and supports the assessment of effectiveness of interventions. To widen the impact and scope, further effort is needed to evaluate the characteristics of the eDNA metabarcoding approach to accurately detect other species using improved annotation databases. With increasing insights into potential weaknesses in biosecurity related to contaminated airborne biological materials, practical interventions could focus on the air coming in through the air inlets, trying to reduce the risk by, for example, air filtering or micro-organism inactivation. Mesh- or filter-based intervention strategies (for instance in airflow heat-exchange equipment) could supplement biosecurity measures to diminish entry of HPAI virus from wild birds into poultry flocks.

## Conclusions

This study demonstrates the entry of wild waterbird DNA into poultry houses through the air inlets, suggesting that airborne HPAI virus could potentially be introduced into poultry houses via the same route. Our eukaryote environmental DNA metabarcoding approach, targeting the actual hosts instead of the pathogen itself, provides a novel tool to monitor, quantify and improve biosecurity measures for pathogens such as HPAI, and provides source attribution modelling possibilities for other pathogens that are difficult to detect at the precise moment of introduction.

## Supplementary Data

866000 Supplementary Material
