# Supplementary material for: Detection of airborne wild waterbird-derived DNA demonstrates potential for transmission of avian influenza virus via air inlets into poultry houses, the Netherlands, 2021 to 2022
Source: Euro Surveill. 2024 Oct 3;29(40):2400350. doi: 10.2807/1560-7917.ES.2024.29.40.2400350 (PMC11451133; doi:10.2807/1560-7917.ES.2024.29.40.2400350)
Supplement: Supplementary Material [file 24-00350_BOSSERS_Supplementary_material.pdf]

Supplementary materials for Bossers *et al.* 2024

DISCLAIMER: "This supplementary material is hosted by *Eurosurveillance* as supporting information alongside the article '**Detection of airborne wild waterbird-derived DNA demonstrates potential for transmission of avian influenza virus via air inlets into poultry houses, the Netherlands, 2021 to 2022**', on behalf of the authors, who remain responsible for the accuracy and appropriateness of the content. The same standards for ethics, copyright, attributions and permissions as for the article apply. Supplements are not edited by *Eurosurveillance* and the journal is not responsible for the maintenance of any links or email addresses provided therein."

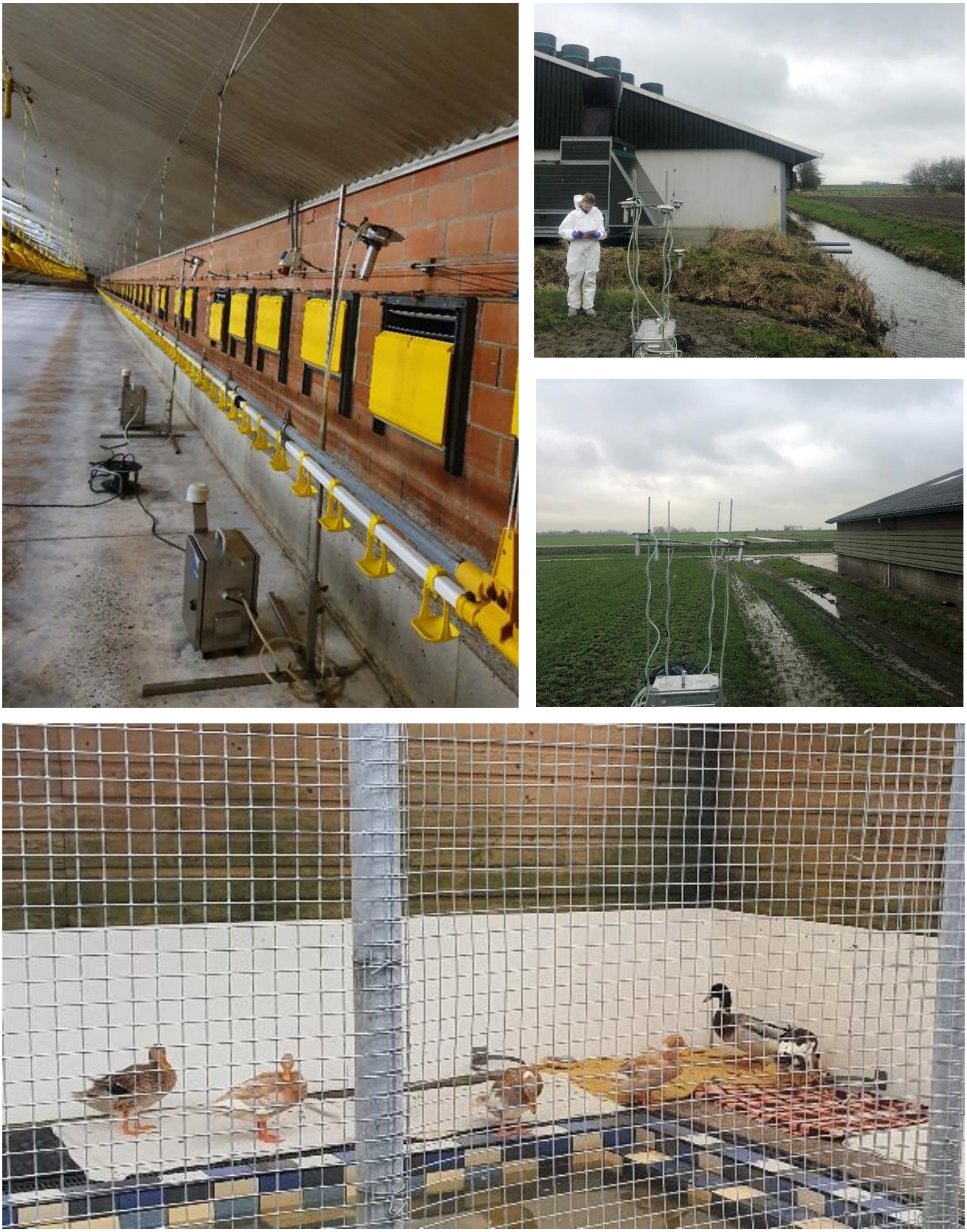

Supplementary Figure S1. Impression of sampling locations. Top-left picture: showing measurement set-up for air sampling inside poultry farm consisting of a sampling head connected to a calibrated pump ensuring active air sampling of 10 L/min (one installation visible in the front and one in the back). Sampling head is directed towards the air-inlet to sample airflow entering the farm. Top-right picture: Protective clothing is worn following applicable biosecurity measures. Middle-right picture: showing measurement set-up for air sampling outside poultry farm consisting of four sampling heads connected to a calibrated pump ensuring active air sampling of 10 L/min. Sampling is performed at approximately 8 meters from the side of the poultry farm, where (not visible because covered by wind breaking panels) air-inlets are present. Bottom picture: one of the bird shelter aviaries where sampling took place

Supplementary Table. Descriptives of the 7 air samples that tested positive for waterbird DNA at the poultry farms.

Farm	Date	Location of sampling
**1 (Broiler)**	10-dec-2021	Inside farm, air inlet orientated to the West side
**2 (Broiler)**	15-jan-2022	Inside farm, air inlet orientated to the South side
	02-feb-2022	Outside farm, at West side
**3 (Layer)**	23-feb-2022	Inside farm, air inlet orientated to the East side
	23-feb-2022	Outside farm, at North-West side
	04-mrt-2022	Outside farm, at South-West side
	09-mrt-2022	Inside farm, air inlet orientated to the West side

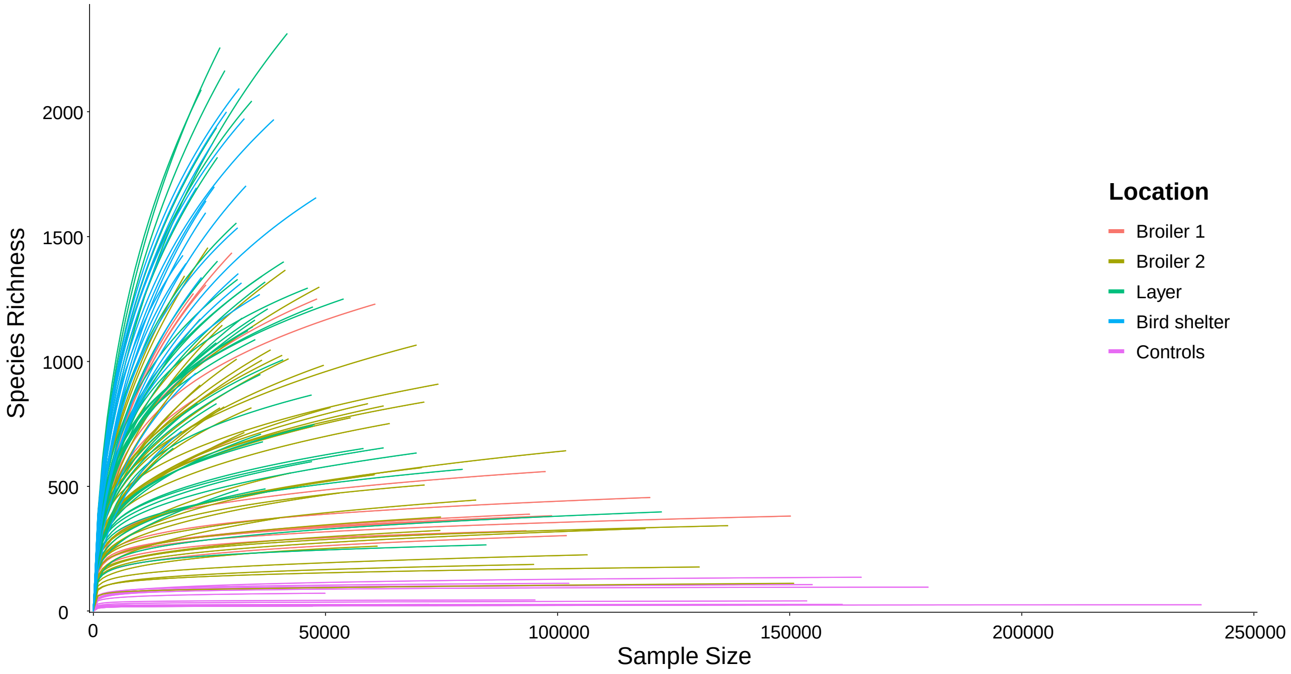

Supplementary Figure S2. Rarefaction curves of the sequenced air samples. The observed number of different species (amplicon sequence variants) per sample are shown as function of the number of subsampled amplicon sequence variants. Different colors represent samples from the various sampling locations or the controls respectively.
